# On the modelling of variance components in classical twin studies

**DOI:** 10.1038/s10038-025-01427-w

**Published:** 2025-11-05

**Authors:** Shobbir Hussain

**Affiliations:** Department of Life Sciences, University of Bath, Claverton Down, Bath, UK

**Keywords:** Genetics, Genomics

## Abstract

Classical twin studies can be used to disentangle the extent to which phenotypic variance of a given complex trait is determined by genetic and environmental variance. The designs widely employ ‘ACDE’ structural equation models where partitioned variances, including that of additive (A) and dominance (D) genetic components, are estimated and where A is taken as reflective of the narrow-sense heritability. Here, it is illustrated in a clear and accessible manner that it is in reality impossible to reliably partition the genetic variance into A and D components using the models which are consequently very open to overestimating the additive genetic variance. This essay should serve as a reminder that classical twin studies can approximate the total (broad-sense) heritability of complex traits, but that leveraging the findings with molecular measurement-based methods is necessary to reliably partition genetic variance components; brief examples based on recent relevant findings is also presented.

The analysis of phenotypic resemblance between human relatives provides means to determine the contribution of genetic variance to phenotypes of interest. Twin studies are particularly useful as they can be performed via simple phenotypic comparisons between monozygotic (MZ) and dizygotic (DZ) twins. By virtue of identity-by-descent, MZ twins will fully correlate for genetic variants while dizygotic (DZ) twins will share on average half of the genetic variants. In the classical twin study design, rates of intra-pair phenotypic correlation for a given trait are compared for MZ and DZ twins thus enabling the relative contributions of genetic and environmental variances to be calculated [1–3], and this classic design still remains by far the most commonly utilised twin study approach [4]. Widely employed frameworks for such analyses are statistical structural equation models (SEMs) based on the ACDE system which aim to partition relative contributions to a phenotype into additive genetic (A), dominance (non-additive) genetic (D), common environment (C), and unique environment (E) variance components [5–7]. Such models aim to enable up to 3 of these variance components (i.e. AE vs ACE vs ADE) to be estimated from a set of twin correlation data, but tend to rely on a number of assumptions; nonetheless, relevant limitations have previously been discussed and the yielded estimates are generally considered as good approximations [5–7]. Thus, use of such models for the analysis and interpretation of relevant twin correlation data is currently considered as best practice in the field [8, 9], and findings from relevant studies have for example been taken as the main empirical indicator that the genetic variance for complex traits in general is likely to be largely additive [10, 11]. Here, specific flaws of the SEM frameworks are more fully illustrated: since the frameworks share a couple of key foundational principles with Falconer’s originally described method [12] for estimating heritability, an understanding of Falconer’s approach can be used to help appreciate the relevant shared limitations.

The following working has been adjusted and simplified for purposes of clarity only; the logic and overall mathematical derivation remains the same as in the original work [12]. Falconer first notes the intra-pair genetic variation coefficients shared by pairs of MZ twins and DZ twins through identity-by-descent probabilities; thus, MZ twins will share between them all of their genetic variation including the additive (1xV_A_) and dominance (1xV_D_) components, while DZ twins will share between them on average half of the additive (½V_A_) and a quarter of the dominance (¼V_D_) variation (since the latter relies on interaction between two genetic variants). Thus, between the two types of twins, the difference in the genetic variation shared is (V_A_ – ½V_A_) + (V_D_ – ¼V_D_) = ½V_A_ + ¾V_D_. It therefore follows that the difference in the rate of intra-pair phenotypic correlation between the two types of twins (rMZ–rDZ) for a given trait will be attributable to the effects of half of the additive and three quarters of the dominance genetic variance for the trait, ½A + ¾D. However, in order that the full genetic variances for the trait can be extrapolated, scaling needs to be applied; since additive variance is generally considered the most important component by geneticists, Falconer opts for a scaling factor of x2 with the incentive of transforming ½A to 1xA, and therefore arrives at:

1 $$2({{{\rm{rMZ}}}}{{{\rm{\hbox{-}}}}}{{{\rm{rDZ}}}})={{{\rm{A}}}}+1\dfrac{1}{2}{{{\rm{D}}}}$$

Note, however, that since rMZ–rDZ = ½A + ¾D, then ideally, we would apply a x2 scaling to the *proportion* of rMZ-rDZ that is explained by A, and a scaling of x ^4^/_3_ to the *proportion* of rMZ-rDZ that is attributable to D, to arrive at the respective individual component genetic variances. To see this more clearly, the ideal approach would be:

2 $$2{{{\rm{y}}}}+\dfrac{4}{3}{{{\rm{z}}}}={{{\rm{A}}}}+{{{\rm{D}}}}$$

where y = proportion of rMZ-rDZ attributable to additive variance, and z = proportion of rMZ-rDZ attributable to dominance variance.

In practice, equation [2] is not useful (and not presented or discussed by Falconer) since the values of y and z are unmeasurable from twin correlation data; nonetheless, the exercise shows that the estimation of individual values for A and D is actually not possible from such experimental designs.

To illustrate this point further, there are further specific non-additive genetic variation terms that are not considered in the working which we can also add in here: DZ twins will also share a quarter of the additive x additive (¼V_AA_), an eight of the additive × dominance (^1^/_8_V_AD_) and a sixteenth of the dominance x dominance (^1^/_16_V_DD_) variation between them (since the variation coefficient shared is 1/2^n^, where n = number of interacting variants). Considering these additional terms as well, we can thus reason that rMZ-rDZ is attributable to the genetic variance components ½A + ¾D + ¾AA + ^7^/_8_AD + ^15^/_16_DD, thus arriving at:

3 $$2({{{\rm{rMZ}}}}-{{{\rm{rDZ}}}})={{{\rm{A}}}}+1\dfrac{1}{2}{{{\rm{D}}}}+1\dfrac{1}{2}{{{\rm{AA}}}}+1\dfrac{3}{4}{{{\rm{AD}}}}+1\dfrac{7}{8}{{{\rm{DD}}}}$$

Again, the ideal approach would instead be to apply the appropriate scaling (x2 for A, x ^4^/_3_ for each of D and AA, x ^8^/_7_ for AD, and x ^16^/_15_ for DD) to the individual proportions of rMZ-rDZ attributable to each of the variance components; such proportions are unmeasurable from twin correlation data thus meaning that respective component values cannot in reality be determined.

Falconer nonetheless presents us with equation [1] (likely since it is the most manageable and suitable available option to estimate heritability), although immediately cautions that the formula does not provide a strictly valid estimate of either narrow-sense (additive only, *h*^*2*^) nor broad-sense (additive + non-additive, *H*^*2*^) heritability, but that it yields an approximation closer to *H*^*2*^ [12]. Note that, hypothetically, it is quite possible for A to be small and thus for 2(rMZ-rDZ) to reflect an estimate largely of 1½D, just as possible for example compared to D being small and thus for 2(rMZ-rDZ) to reflect an estimate largely of A, or anything in between; there is nothing within Falconer’s formula nor within the mathematical derivation of it that suggests that one possibility should be favoured over the other. Thus, it is not possible in practice, irrespective of the sophistication of any statistical modelling that might be applied, for values of A and D to be estimated from the system–only their sum can actually be approximated.

The above discussion is also directly relevant to SEM frameworks commonly used today for such heritability calculations, since although they employ sophisticated maximum likelihood estimation methods with good statistical properties, they are nonetheless based on the strong assumption that 2(rMZ–rDZ) yields an estimate of A while acknowledging that this will be overestimated by a value of 1½D if non-additive effects are incorrectly ignored [13]. Thus, in such modelling, all of the genetic variance will be blindly attributed to A when rMZ<2rDZ or rMZ = 2rDZ [5–7]. If the condition rMZ > 2rDZ is observed, then as much of the estimated genetic variance as possible is similarly attributed to A, and the remainder attributed to D [5–7]. Therefore, in instances where either rMZ < 2rDZ or rMZ = 2rDZ, and where dominance does in reality play at least some role, then the ACDE model-derived values for A will in fact truly be reflective of an overestimate of the broad-sense heritability (an overestimate since the 1.5 x over-scaling of D will not have been accounted for). The extent to which A is overestimated, and D thus underestimated, in such instances is unknown, but such potential mis-estimations could be very significant. Similar concerns apply to when rMZ>2rDZ; here values for both A and D are calculated from the model, but in all cases the extent to which A may be overestimated, and D thus underestimated, is unknown, and if D in reality plays a larger role than assumed, then the sum value A + D will further represent at least some overestimate of the broad-sense heritability. Thus, it is apparent that in general a much less-assumptive representation of relevant heritability estimates yielded from twin studies is actually ‘*H*^*2*^*’* (and not ‘*h*^*2*^*’* as very frequently presented); however, since these might often be overestimates, then something akin to ‘*H*^*2*^_*twin*_’ is likely more appropriate. But the most important point to take away from the above discussions is that we see that individual values for A and D cannot actually be measured from twin correlation data, and due to foundational principles inherent to their construction, the widely employed SEM frameworks will have a propensity to overestimate A while underestimating D (and/or other non-additive components). Thus, for example, observations of twin correlation data across numerous traits frequently being found to be best-fitting with the condition rMZ=2rDZ [10, 11] may not necessarily in reality be reflective of a generally additive genetic architecture for such traits.

The likely importance of the above-described problems may be further illustrated by considering estimations of heritability of traits using recent genomics-based methods [14, 15]. We can thus consider here relevant observations made for height and body mass index (BMI); although many other suitable complex traits and biological measurements could in principle be considered, the large sample sizes required for the relevant genomic studies [14, 15] have so far meant that according reliable studies only for BMI and height have thus far been performed. Thus, GREML-based whole genome sequencing (WGS)-heritability estimates are ~0.7 for height and ~0.3 for BMI, and such estimates likely provide very close estimations of their total narrow-sense heritabilities [14]. Therefore, the twin study heritability (*H*^*2*^_*twin*_) for height of ~0.8 is almost fully recovered by its narrow-sense heritability, while the same cannot be said for BMI—the twin study heritability (*H*^*2*^_*twin*_) of which stands at ~0.7 [2, 3]. This suggests that non-additive genetic effects may be a very significant variance component for BMI. This starkly contradicts twin study ACDE model-based partitioning of BMI variance components, where large scale meta-analyses suggest that the calculated heritability of around 0.7 is virtually exclusively additive [16–21]. Given the large discrepancy, we may thus also consider findings of a very recent genomics-based identity-by-descent (IBD) study that calculated the heritability of height at ~0.75 and that for BMI at ~0.55 [15]. Full-sibling pairs were analysed in the study and therefore the effects of any non-additive as well as additive components would be captured in these heritability calculations, and in general such estimates may be taken as approximating broad-sense heritability [22]. Comparing the WGS-heritability-based narrow-sense heritability estimate of ~0.3 and the full sibling-IBD-based broad-sense heritability estimate of ~0.55 for BMI, again, nonetheless suggests that a very significant proportion of the genetic variance for the trait is non-additive. This view is yet further supported via IBD analysis in more distantly related individuals, where non-additive genetic effects would be much more dilute, thus yielding heritability estimates much closer to the narrow-sense heritability; here, the heritability was also calculated at ~0.3 for BMI [23], thus further raising the possibility that the gap to the full sibling-IBD-based heritability of ~0.55 might be due to non-additive variance. It is also worth noting that, given the observation of full sibling-IBD-based heritability being estimated at ~0.55, the twin study heritability (*H*^*2*^_*twin*_) calculation for BMI of ~0.7 may be overestimated; as described in the sections above, such inflation could at least in part be accounted for by additive variance overestimation coupled with non-additive variance underestimation. Thus, for traits such as height where the genetic architecture is indeed in reality explained almost exclusively by additive variance, the respective twin study heritability estimates and ACDE model-based partitioning of such are likely faithful; however, as exampled with BMI, other types of traits may not follow this pattern.

With the application of newly developed molecular measurement-based methods to estimate the heritability of complex traits [14, 15], we may be headed toward a crossroads in our views of their genetic architecture. This essay presents a timely reminder of the potential pitfalls of the reliance on heritability estimates yielded from classical twin studies [4, 24]; it may be helpful to more generally view the estimates as approximations of broad-sense heritability with less emphasis on attempting to partition genetic variance components, as it is possible that the latter may frequently lead to erroneous outcomes and thus ultimately to inadvertently misleading perceptions.
